# New Proteomic Signatures to Distinguish Between Zika and Dengue Infections

**DOI:** 10.1016/j.mcpro.2021.100052

**Published:** 2021-02-12

**Authors:** Kristina Allgoewer, Shuvadeep Maity, Alice Zhao, Lauren Lashua, Moti Ramgopal, Beni N. Balkaran, Liyun Liu, Savita Purushwani, Maria T. Arévalo, Ted M. Ross, Hyungwon Choi, Elodie Ghedin, Christine Vogel

**Affiliations:** 1 Department of Biology, Center for Genomics and Systems Biology, New York University, New York, New York, USA; 2 Department of Biology, Humboldt University, Berlin, Germany; 3 Clinical Research Division, Martin Health System, Stuart, Florida, USA; 4 Department of Clinical Medical Sciences, University of the West Indies, St Augustine, Trinidad & Tobago; 5 Center for Vaccines and Immunology, University of Georgia, Athens, Georgia, USA; 6 Department of Infectious Diseases, University of Georgia, Athens, Georgia, USA; 7 Department of Medicine, Yong Loo Lin School of Medicine, National University of Singapore, Singapore; 8 Department of Epidemiology, School of Global Public Health, New York University, New York, New York, USA

**Keywords:** proteomics, Zika, dengue, flavivirus, infectious disease, data-independent acquisition, serum, mass spectrometry, CDC, Center for Disease Control, DDA, data-dependent acquisition, DENV, dengue virus, DIA, data-independent acquisition, ELISA, Enzyme-Linked Immuno-Sorbent Assay, FGA, Fibrinogen Alpha, NAAT, nucleic acid amplification test, PF4V1, Platelet Factor 4 Variant 1, PPBP, Pro-Platelet Basic Protein, PRNT, Plaque or Focus Reduction Neutralization Test, QC, quality control, RT, retention time, ZIKV, Zika virus

## Abstract

Distinguishing between Zika and dengue virus infections is critical for accurate treatment, but we still lack detailed understanding of their impact on their host. To identify new protein signatures of the two infections, we used next-generation proteomics to profile 122 serum samples from 62 Zika and dengue patients. We quantified >500 proteins and identified 13 proteins that were significantly differentially expressed (adjusted *p*-value < 0.05). These proteins typically function in infection and wound healing, with several also linked to pregnancy and brain function. We successfully validated expression differences with Carbonic Anhydrase 2 in both the original and an independent sample set. Three of the differentially expressed proteins, *i.e.*, Fibrinogen Alpha, Platelet Factor 4 Variant 1, and Pro-Platelet Basic Protein, predicted Zika virus infection at a ∼70% true-positive and 6% false-positive rate. Further, we showed that intraindividual temporal changes in protein signatures can disambiguate diagnoses and serve as indicators for past infections. Taken together, we demonstrate that serum proteomics can provide new resources that serve to distinguish between different viral infections.

## Highlights

• Analysis of human serum samples with extreme protein abundance ranges
• Unique protein signatures for Zika and dengue virus infection
• Temporal changes in protein signatures as indicators for past infections
• Machine learning to account for confounding factors

## Graphical abstract

**Figure:**
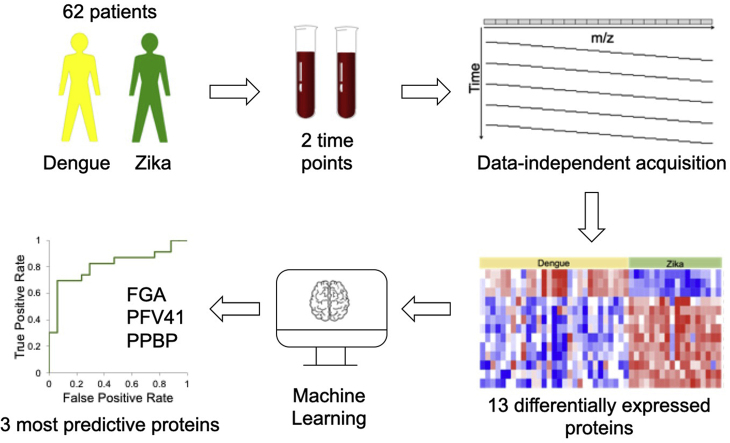

## In Brief

Differentiation between the mosquito-borne Zika and dengue Flavivirus infections is clinically important for correct treatment, but remains challenging. We used mass spectrometry to quantify expression levels for 277 proteins measured in serum samples from 62 patients, providing a resource to the community. We identified 13 proteins with significant differential expression between the closely related types of infections. Most of the proteins link to pregnancy and brain function. We also identified expression signatures that mark ambiguous infections with respect to temporal differences.

Zika virus (ZIKV) and dengue virus (DENV) are closely related flaviviruses transmitted by the same mosquito vector, *Aedis aegypti*, and with overlapping geographical distributions (1, 2). While most ZIKV and DENV infections are asymptomatic, they cause a similar immune response and symptoms in the host, including fever and body pain (1, 2). In contrast to DENV, ZIKV infections in pregnant women pose a significant risk to the developing embryo, with microcephaly and other adverse outcomes (3, 4, 5).

In some geographic areas, diagnosis is purely based on symptoms and endemicity of the virus, which leads to problems due to the shared febrile syndrome. Other affected regions use molecular tests, such as nucleic acid amplification tests (NAATs) of the viral RNA. NAATs are recommended <7 days after onset of symptoms (6). They are highly sensitive and specific, but RNA extraction can be difficult due to sample instability. Therefore, NAATs might present false-negative results, in particular if the sample was collected more than 7 days after onset of symptoms (7).

For symptomatic patients with negative NAAT results or where serum was collected more than 7 days after onset of symptoms, diagnosis should be complemented by antibody-based tests that include Enzyme-Linked Immuno-Sorbent Assay (ELISA), Plaque or Focus Reduction Neutralization Tests (PRNTs), or rapid antigen testing. IgM antibodies typically develop during the first week of illness, but little is known about IgM longevity following infection (6). Neutralizing antibodies, such as IgG, develop shortly after IgM antibodies arise and persist for many years after an infection.

DENV infection is typically diagnosed through IgG and IgM tests in conjunction with geographic location and patient symptoms. IgM antibody tests typically result in more false-positives than NAATs due to nonspecific reactivity or cross-reactivity with other flaviviruses. For example, dengue-virus-reactive T cells are thought to potentially mediate cross-protection against subsequent ZIKV infections (1).

According to guidelines of the Center for Disease Control (CDC), positive IgM antibody tests with negative NAAT results should be confirmed by neutralizing antibody tests when clinically or epidemiologically indicated (6). Neutralizing antibody tests are performed by PRNTs to measure antibody titers for dengue, Zika, and other flaviviruses. PRNTs can resolve false-positive IgM antibody results caused by nonspecific reactivity and, in certain cases, can help identify the infecting virus. In primary flavivirus infections, a neutralizing antibody titer ≥4-fold higher than titers against other flaviviruses to which the person might have been exposed usually determines the specific infecting flavivirus (6). While neutralizing antibody titers might be able to differentiate dengue and Zika virus infections, particularly in specimens collected ≥3 months after illness onset, a fourfold higher titer by PRNT in one *versus* the other infection might not discriminate between the two illnesses during the acute symptoms, especially following secondary flavivirus infections. Consequently, in areas with high prevalence of dengue and Zika virus infections, PRNT might not define the infecting virus for a significant proportion of cases (6, 8). Absence of positive DENV testing in the presence of other symptoms, including pain behind the eyes, often leads to ZIKV diagnosis.

While antibody-based testing is an important diagnostic tool, interpretation of the results is complicated by cross-reactivity of the IgG antibodies leading to false-positives (6, 9, 10, 11). Similar cross-reactivity challenges rapid antigen testing (12). IgM antibodies are specific enough to distinguish between Zika and dengue infections, but appear only early in infection. In addition, previous infections can impact the assumed time point of the current infection due to IgG antibody longevity. For example, in persons previously infected with, or vaccinated against, a Flavivirus, subsequent infection with another Flavivirus can result in both a diminished IgM response and a rapid increase in neutralizing antibodies against multiple Flaviviruses, which might preclude conclusive determination of which virus was responsible for the person’s most recent infection (6). The timing and presence of virus-specific anti-IgM and -IgG antibodies are therefore insufficient to distinguish between the two infections, in particular in areas where both viruses are endemic. These challenges are particularly critical in pregnant women suspected of ZIKV infections, and the CDC recommends to consider epidemiological data on viruses circulating at the location of exposure and clinical symptoms when diagnosing Zika and dengue virus infections (6).

The short diagnostic window for virus-specific tests combined with the extensive serologic cross-reactivity highlights the continued need to understand the impact of DENV and ZIKV infections on the host (7). However, several additional factors complicate sample analysis even further. Samples are often collected from diverse sites without standardized protocols or note of associated patient data, and sample cohorts are typically very small (7). Further, blood samples can rapidly change in composition depending on storage and processing times. Finally, proteins in serum have enormous abundance differences, *e.g.*, the concentration of serum albumin is an order of magnitude larger than that of other proteins. Even most advanced proteomics studies struggle to identify more than a few 100 proteins in large-sample analysis (13, 14). The problem is exacerbated in studies that struggle with obtaining a sufficient amount of proteins or those in which samples are collected under suboptimal conditions.

To address these challenges and provide new resource for the scientific community to understand the proteomic response to DENV and ZIKV infections, we used state-of-the-art mass-spectrometry-based methods that are particularly amenable to protein mixtures with extreme dynamic ranges (15) and analyzed a cohort of patients with DENV or ZIKV infections from Trinidad. We subjected the data to rigorous statistical modeling to remove confounding effects as much as possible and identify differentially expressed proteins. Most of the differentially expressed proteins have links to pregnancy and brain function. We also discuss proteomic signatures of patients with ambiguous diagnosis, which may, in the future, contribute to identifying past infections as well as coinfections.

## Experimental Procedures

### Experimental Design and Statistical Rationale

We obtained 124 serum samples from a Trinidadian cohort of 62 patients diagnosed with either DENV or ZIKV infections in 2016 and 2017. For 61 of those patients, samples had been taken at two time points several days apart. Both samples from one of the patients had to be removed due to lack of protein, leaving 122 samples from 62 patients for our subsequent analysis. Of those, 68 had been classified as DENV samples and 54 as ZIKV samples. Female and male patients were described as either Afro-Trinidadian or Indo-Trinidadian, aged between 23 and 51 years. Patients were similarly distributed with respect to gender, ethnicity, and age.

The order of proteomics samples was randomized prior to the mass spectrometry run using the rand function in Excel. Data was acquired in data-independent acquisition (DIA) mode as described below in batches of 4 to 8 samples. A quality control (QC) sample derived from the sample pool was run in between batches, resulting in 20 QC samples. The sample pool was created from ten randomly chosen samples. After our filtering, QC, and fragment quantification, samples were split into training and test sets (details below). Using the training set, differentially expressed proteins were identified by multiple linear regression and the most predictive proteins by logistic regression. Results were then evaluated with the test set. A spectral library was created from data-dependent acquisition mass spectrometry analysis of 20 fractions of the sample pool.

To estimate power of the analysis, an effect size of 0.5, power of 0.8, and significance level of 0.05 would require 128 samples with equal distribution between DENV and ZIKV patients. The cohort analyzed here roughly meets these criteria (122 samples, 68 DENV, and 54 ZIKV patients’ samples). However, as we quantified 277 proteins (see below), we would need to adjust the significance level to 0.00017 to account for multiple hypothesis testing. This significance level in turn would require 346 samples. Supplemental Fig. S3 in the Supplementary Material shows the effect sizes of the clinical data for different principal components.

### Diagnostic Testing

Diagnosis of DENV or ZIKV infections by Trinidadian doctors was based on positive or negative anti-DENV IgM/IgG results using a rapid, dual IgM/IgG test (Panbio Dengue Duo Cassette) and the presence or absence of symptoms such as headache, pain behind the eyes, vomiting, fever, body pain, and rash. The three symptoms (headache, pain behind eye, and vomit) were presumably used by local doctors to distinguish ZIKV from DENV infections. Supplemental Table S1 lists meta-data for all samples.

The Institutional Review Boards at the University of Puerto Rico, the Centers for Disease Control and Prevention, and University of Georgia approved this study, #00003640. Written informed consent and assent, where appropriate, were obtained from all participants (or their parents/legal guardians) prior to beginning study procedures.

Supplemental Table S6 lists results for additional serology tests (at a later point for a subset of samples) with antibodies against ZIKV IgM and in Focus Reduction Neutralization Tests (FRNT) for ZIKV and DENV 1 to 4. The Zika IgM antibody capture enzyme-linked immunosorbent assay was conducted as follows. Anti-IgM (the capture antibody) was coated on 96-well plates. Patient serum was diluted 1:400 and allowed to bind. Viral antigen (Zika Virus MR766 culture from Vero cells) was added and allowed to bind. The presence of Zika viral antigen was detected by using HRP-conjugated anti-Zika E antibody. A colorimetric result was generated by the interaction of the enzyme and an Enhanced K-Blue TMB substrate. This colorimetric change was detected by a spectrophotometer (ELISA reader). The FRNT assays for DENV 1 to 4 using prototype viruses and adapted for Zika using MR766 virus were conducted as previously published (16). The infecting DENV strain was identified in some of the samples where FRNT were performed. Traditionally, a fourfold higher FRNT titer of one virus over another indicates the current virus. However, it is now accepted that this may still not help discriminate between viruses, during acute illness, and especially following secondary infections in endemic areas (https://www.cdc.gov/zika/comm-resources/infographics.html).

The Pan American Health Organization reports total incidents for Trinidad/Tobago as dengue: 2014: 128; 2015: 1687; 2016: 1801; 2017: 300; 2018: 123; and Zika: 2014: na; 2015: 0; 2016: 722; 2017: na; 2018: na (https://www.paho.org).

### Proteomics Sample Preparation

We resuspended serum samples (∼75 μg) with 0.1% Rapigest (Waters) in 100 mM ammonium bicarbonate (Sigma-Aldrich) and incubated for 5 min at 95 °C to facilitate protein denaturation. It was then reduced with 5 mM dithiothreitol (Sigma-Aldrich) for 30 min at 60 °C, followed by alkylation with 15 mM iodoacetamide (Sigma-Aldrich) at room temperature for 30 min in the dark. We digested the samples overnight using sequencing grade modified porcine trypsin (w/w ratio 1:50) (Sigma-Aldrich) on a thermomixer at 37 °C, 200 RPM (Eppendorf). Rapigest surfactant was cleaved by incubating samples with ∼200 mM HCL (Sigma-Aldrich) for 30 min at 37 °C. We desalted digested protein samples on C18 spin tips (Thermo Fisher Scientific) and dried the peptides under vacuum. The dried peptides were resuspended in 5% acetonitrile, 0.1% formic acid (Sigma-Aldrich). The peptide concentration was measured using a fluorometric peptide quantification kit (Thermo Fisher Scientific).

For the mass spectrometry analysis, we constructed a pooled QC sample, which contained aliquots of ten randomly chosen samples. To construct the spectral library, we pooled aliquots from all 124 samples and fractionated the mixture using high-pH reversed-phase high-performance liquid chromatography on an Agilent 1200 Infinity Series HPLC with a phenomenex Kinetex 5 u EVO C18 100A column (100 mm × 2.1 mm, 5 mm particle size). Mobile phase A contained 20 mM ammonium formate, and B contained 90% acetonitrile and 10% 20 mM ammonium formate. Both buffers were adjusted to pH 10. Peptides were fractionated using a linear 70 min 0 to 40% acetonitrile gradient at a 100 μl/min flow rate. Eluting peptides were collected into 2 min fractions. We combined fractions to 20 samples for mass spectrometric analysis. The volume of recombined fractions was reduced using an Eppendorf Concentrator Vacufuge Plus and suspended in HPLC-grade water containing 5% acetonitrile and 0.1% formic acid.

### Mass Spectrometry

We used an EASY-nLC 1000 coupled online to a Q Exactive High Field mass spectrometer (both Thermo Fisher Scientific) for chromatography and mass spectrometry, respectively. Buffer A (0.1% formic acid in water) and buffer B (80% acetonitrile, 0.1% formic acid) were used as mobile phases for gradient separation. Separation was performed using a 50 cm x 75 μm i.d. PepMap C18 column (Thermo Fisher Scientific) packed with 2 μm, 100 Å particles and heated to 55 °C. We used a 155 min segmented gradient of buffer A to buffer B at a flow rate of 250 nl/min as follows: 2 to 5% buffer B for 5 min, 5 to 25% buffer B for 110 min, 25 to 40% buffer B for 25 min, 49 to 80% buffer B for 5 min, and 80 to 95% buffer B for 5 min. Buffer B was held at 95% for another 5 min.

Supplemental Table S1*A* details the order of sample runs for data-DIA. All samples were analyzed as follows: a full-scan MS was acquired in the Orbitrap with a resolution of 120,000, scan range of 350 to 1650 m/z, maximum injection time of 100 ms, and an Automatic Gain Control (AGC) target of 3e6. Subsequently, 17 DIA variable windows were acquired in the Orbitrap with a resolution of 60,000, AGC target of 1e6, and maximum injection time in auto mode. The variable window sizes are listed in supplemental Table S1*B*. We acquired about 4 to 6 data points per peak (average of 5).

For the spectral library, we analyzed the fractionated samples in data-dependent acquisition (DDA) mode. Full MS scans were acquired with a resolution of 120,000, an AGC target of 3e6, a maximum injection time of 100 ms, and scan range of 375 to 1500 m/z. Following each full MS scan, data-dependent high-resolution HCD MS/MS spectra were acquired with a resolution of 30,000, AGC target of 2e5, maximum injection time of 50 ms, 1.5 m/z isolation window, fixed first mass of 100 m/z, and NCE of 27 with centroid mode.

Further details for the mass spectrometry data acquisition are provided with the deposited data.

### Computational Processing of the Proteomics Data

All 144 DIA samples consisting of 124 patient and 20 QC samples were analyzed using Spectronaut (Software Version: 12.0.20491.12, https://biognosys.com/shop/spectronaut) against the project-specific spectral library using default settings. Two samples failed in their data acquisition and were removed from the analysis. XICs were extracted based on the internal retention time (RT) calibration provided by Spectronaut. The average RT extraction window width determined by Spectronaut was in the range of 13.19 min for all runs of this experiment (after removal of faulty samples). No retention time alignment was used between runs. Peak selection and identification were based on Spectronauts proprietary peak detection and scoring algorithms. Spectronaut performs an internal mass calibration with optimized mass tolerance prediction on a per peptide basis. The average PPM tolerance applied for data extraction was in the range of 5.15 and 9.73 ppm for MS1 and MS2, respectively (after removal of faulty samples). False discovery rate (FDR) was calculated run-wise at the peptide precursor and experiment wide at the protein group level and filtered for 1% at both levels. The FDR calculation in Spectronaut is based on mProphet (17).

The spectral library based on the DDA data was prepared using the Pulsar search engine within Spectronaut with default settings that included Trypsin/P digest, peptide length of 7 to 52 amino acids and up to two missed cleavages. The FASTA file for human was downloaded from UniProt on 2/15/2018 and contained 93,798 entries including protein isoforms. Fixed modifications included cysteine carbamidomethylation. Variation modifications included acetylation on the N terminus and methionine oxidation. Pulsar performs by default an internal mass calibration with optimized mass tolerance prediction for precursor and fragment ions. The FDR was calculated by Pulsar for peptide-spectrum matches, peptides, and protein groups and filtered for 1% at all three levels.

We used in-house R scripts to process Spectronaut output, *e.g.*, with respect to deriving a unique fragment identifier and removing replicate fragment ions. We removed 21 proteins with single-peptide identifications. We also removed fragment entries with <50 intensity and any faulty samples (*e.g.*, both samples of patient 48 that did not contain protein). We filtered for fragments with <40% missing values across samples. We removed signal drift in the analysis sequence and batch effect caused by replacement of the chromatographic column by fitting lowess curve (bandwidth parameter f = 0.25 in lowess function in R) for each fragment and subtracting the fitted value from each sample within each batch (supplemental Fig. S1). We then set the average to a common overall average peak area value in the unadjusted data. We then removed all values that remained three or more standard deviations away from the median for outlier removal. This procedure resulted in 277 proteins with high-quality quantitation, based on a minimum of one peptide per protein and at least three fragments per peptide (supplemental Table S2). Supplemental Fig. S2 shows the coefficient of variance (cov) for fragment intensities after batch correction. As we had used several other stringent filters (see above), we did not apply additional filtering by cov.

Principal component analysis was conducted with the prcomp() function using log base 2 transformed and mean-centered expression data with missing values imputed with the impute.knn function with default settings. Supplemental Table S3 shows the correlation of the principal components with meta-data at the fragment and protein level. We then used mapDIA (4) to derive protein-level abundance values from fragment ion intensities and added 2000 counts to all ion counts to prevent spurious overestimation of fold changes in low-abundance proteins (supplemental Table S2).

### Identification of Differentially Expressed Proteins and Development of Predictive Tools

After the removal of two samples without protein content, we split the data set (122 samples) into a training set (67%, 82 samples) and test set (33%, 40 samples). To discover differentially expressed proteins, we devised a two-step analysis. For Analysis A, we used in-house R-scripts (https://www.r-project.org/) to develop a linear regression model for the 82 samples in the training set. The lm() function regressed diagnosis type over the log base 2 transformed protein abundance values and resulted in *p*-values associated with each protein for its association. Benjamini–Hochberg correction for multiple hypothesis testing (18) identified three proteins as differentially expressed with respect to the diagnosis (adjusted *p*-value < 0.05).

For Analysis B, we used the same in-house R-scripts on a subset of 47 samples from the training set for which information on possible confounding factors was present (IgM dengue, Gender, Race, Days since onset, Age). We excluded the anti-DENV IgG information due to its high correlation with the anti-DENV IgM data (identical status). We subtracted the median values for continuous variables (Age, Days_since_onset) to ensure consistent scaling. We employed the multiple linear regression model *via* the lmb() function, including the confounding factors. After Benjamini–Hochberg correction, Analysis B resulted in 11 proteins with statistically significant differences between samples from DENV- or ZIKV-infected patients (adjusted *p*-value < 0.05). We considered the union of Analysis A and B, resulting in 13 proteins, for all subsequent analysis. Supplemental Table S4 lists the results of Analyses A and B, supplemental Table S5 lists details for the differentially expressed proteins.

Two of the 13 differentially expressed proteins were quantified by only one peptide (CA2 and FGG). To confirm their correct identification, we mapped the respective peptide against a large sequence database using blastp (https://blast.ncbi.nlm.nih.gov/Blast.cgi). In both cases, the peptide–protein match was the strongest hit identified (*not shown*). Further, we examined the intensity distributions for the fragments quantifying the two proteins, and they show more consistent patterns than the distribution for fragments of a protein not significantly differentially expressed (supplemental Fig. S4).

The heatmap in Figure 3*A* shows the expression patterns for the 13 significantly differentially expressed proteins. Clustering was performed with the hclust() function with default settings. Heatmaps were produced with the pheatmap() function.

We repeated the analyses constructing training and test sets based on patients (*not shown*). The results confirmed the analysis above: we identified 13 significant proteins of which 12 were identical to those from our sample-based analysis (identifying Transthyretin instead of Gelsolin).

We used the normalized protein abundance counts for the 13 proteins with significant expression differences between DENV and ZIKV cases to predict the diagnosis for the independent test set of 40 samples. We used the WEKA machine learning environment (19) (https://sourceforge.net/projects/weka/) and tested various built-in algorithms for performance. Within WEKA, CFS Subset selection with the “Best first” search method resulted in four proteins with the highest predictive value: P02671 (FGA), P02775 (PPBP), P06396 (GSN), and P10720 (PF4V1). We then developed different classifier models on the training set and evaluated performance in the independent test set. The best result was obtained using Logistic Regression with Fibrinogen Alpha (FGA), Pro-Platelet Basic Protein (PPBP), Platelet Factor 4 Variant 1 (PF4V1). Using tenfold cross-validation in WEKA Experimenter, the percentage of correct predictions could be improved using the parameters -R 6.0 -M -1 (not significant). We used the final classifier model on the independent test set to calculate true- and false-positive rates (Fig. 3*C*). Changing the parameters had no effect on the percentage of correct predictions in the test set.

All R-scripts and the final classifier are deposited as supplemental Files.

### Further Validation and Characterization of Predictions

We evaluated the diagnostic potential of some proteins conducting western blots with anti-FGA, anti-FGG, anti-CA2 antibodies. The quality of anti-FGB and anti-PF4V1 antibodies was insufficient for further analysis. We conducted all western blots in samples from both the original 2016/2017 and cohort obtained in 2017/2018 (supplemental Fig. S8). The 2017/2018 samples had been collected independently, following the same procedures as described for the 2016/2017 cohort used for the proteomics studies described here.

We extracted proteins from serum samples using the following procedure. The serum aliquots were diluted and sonicated for 12 s at 8 amp with a 30 s interval on ice. We added ice-cold acetone and trichloroacetic acid in a vol/vol ratio of 1:8:1 (sample:acetone:trichloroacetic acid). The samples were then mixed and kept at −20 °C for 1 to 5 h. Samples were then centrifuged at 18,000*g* for 15 min at 4 °C in a micro centrifuge. We discarded the supernatant and washed the pellet with 1 ml ice-cold acetone. Samples were centrifuged again at 18,000*g* for 15 min at 4 °C. This wash step was repeated another two times.

We dried the remaining pellet at room temperature and suspended the protein in sodium dodecyl sulfate buffer (20 mM EDTA, 140 mM NaCl, 5% SDS, and 100 mM Tris pH 8.0.). We estimated protein concentrations using the Pierce BCA kit (Thermofisher scientific). Equal amounts of protein (30 μg) from individual samples were subjected to western blotting. The membrane was blocked using 5% BSA and incubated with respective antibodies (rabbit anti-FGA antibody (1:2000, Abcam Cat no: ab92572), rabbit anti-FGG antibody (1:1000, Abcam Cat no: ab62527), rabbit anti-CA2 antibody (1:2000 Abcam Cat no: ab191343)). Ponceau staining served as a loading control. We captured signal intensities of the bands in the western blot with Kwikquant Imager (Kindle Biosciences). Quantitative results are available in supplemental Table S7.

We examined mispredictions (*i.e.*, discrepancies between the original diagnosis and the predicted diagnosis) in more detail to understand if mispredictions arose from intrinsic sample properties such as multiple infections or ambiguous diagnosis. To do so, we used information from additional serological testing described above. Further, we used the two-step analysis (Analysis A and B) to screen for proteins differentially expressed between unambiguously and ambiguously diagnosed patients.

## Results

### A Cohort of Patients with DENV and ZIKV Infections Provided Complex Meta-Data

**Fig. 1:**
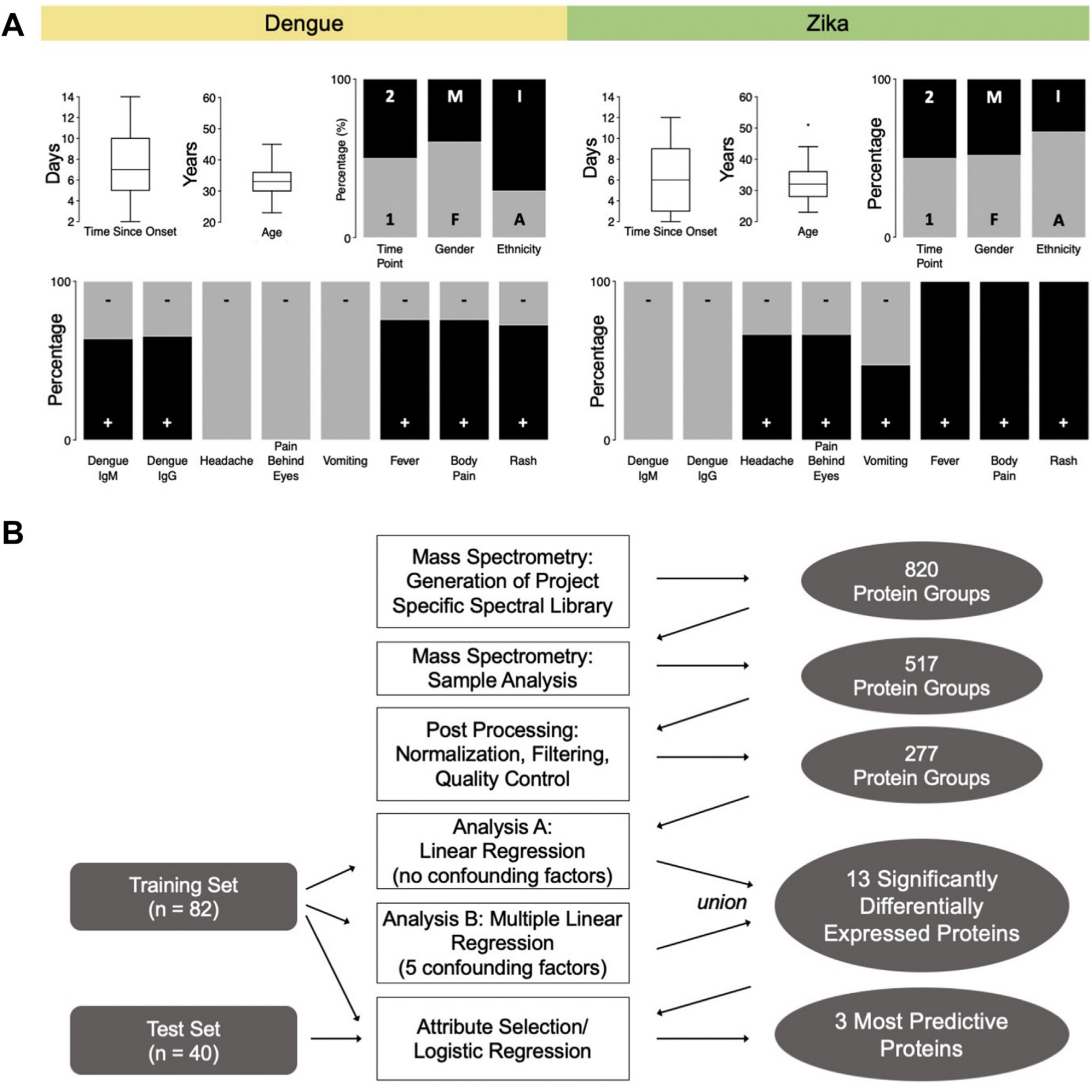
**Patients with DENV or ZIKV infection are from comparable backgrounds.***A*, we collected 122 serum samples from 35 and 27 patients with DENV or ZIKV infection, respectively. The samples were taken at two time points after onset of symptoms: 3 to 7 days for the first time point (1), 7 to 14 days for the second time point (2). Female (F) and male (M) patients were described as either Afro-Trinidadian (A) or Indo-Trinidadian (I), aged between 23 and 51 years. Diagnosis of DENV or ZIKV infection by local doctors was based on a positive (+) or negative (−) anti-DENV IgM/IgG ELISA result and the presence or absence of headache, pain behind the eyes, vomiting, fever, body pain, and rash. *B*, the proteomics and statistical workflow includes: generation of a project specific spectral library of 820 protein groups, detecting 517 protein groups, and retaining 277 protein groups after normalization, filtering, and quality control steps. We then used a two-step analysis (Analysis A and B) to identify a total of 13 differentially expressed proteins (adjusted *p*-value < 0.05). We extracted three most predictive proteins and evaluated their predictive ability.

Using high-resolution mass spectrometry, we screened 122 serum samples collected in 2016 and 2017 from a cohort of 62 patients with DENV or ZIKV infections (Fig. 1, supplemental Table S1). The patients had been recruited from emergency departments in Trinidad. Samples were taken at two different time points after onset of symptoms, *i.e.*, 3 to 7 and 7 to 14 days for time points 1 and 2, respectively. For two patients (80 and 81), only one time point had been collected. Patients had been diagnosed in Trinidad based on commercial diagnostics against DENV IgM and IgG (Methods) and symptoms. We used this original diagnosis as the “gold standard” for evaluation of the new proteomics data. The additional serological testing (Methods) revealed limitations of these diagnoses, as discussed below.

Patients from the two infection types were similarly distributed with respect to gender, ethnicity (Afro-Trinidadian or Indo-Trinidadian), and age (23–51 years) (Fig. 1). The symptoms (headache, pain behind the eyes, vomiting, fever, body pain, rash) showed biases between the patient groups, but not exclusive classification. Three of the symptoms were only diagnosed in patients with ZIKV and absent in patients with DENV: headache, pain behind the eyes, and vomiting.

Figure 1 also shows that current diagnostic markers have several inconsistencies. Typically, if patients tested negative in the DENV IgM/IgG ELISA, they were diagnosed as having Zika (supplemental Table S1). However, nine patients (84, 86, 87, 88, 89, 90, 91, 97, 98) were diagnosed as having DENV, even though positive DENV IgG/IgM were not reported. Patient samples 79 to 101 had originally missing IgM/IgG results, but were collected during a DENV infection outbreak, and for some samples additional serology provided a diagnosis (supplemental Table S6).

### Quantitative Proteomics Revealed Diverse Expression Patterns Across Patients

**Fig. 2:**
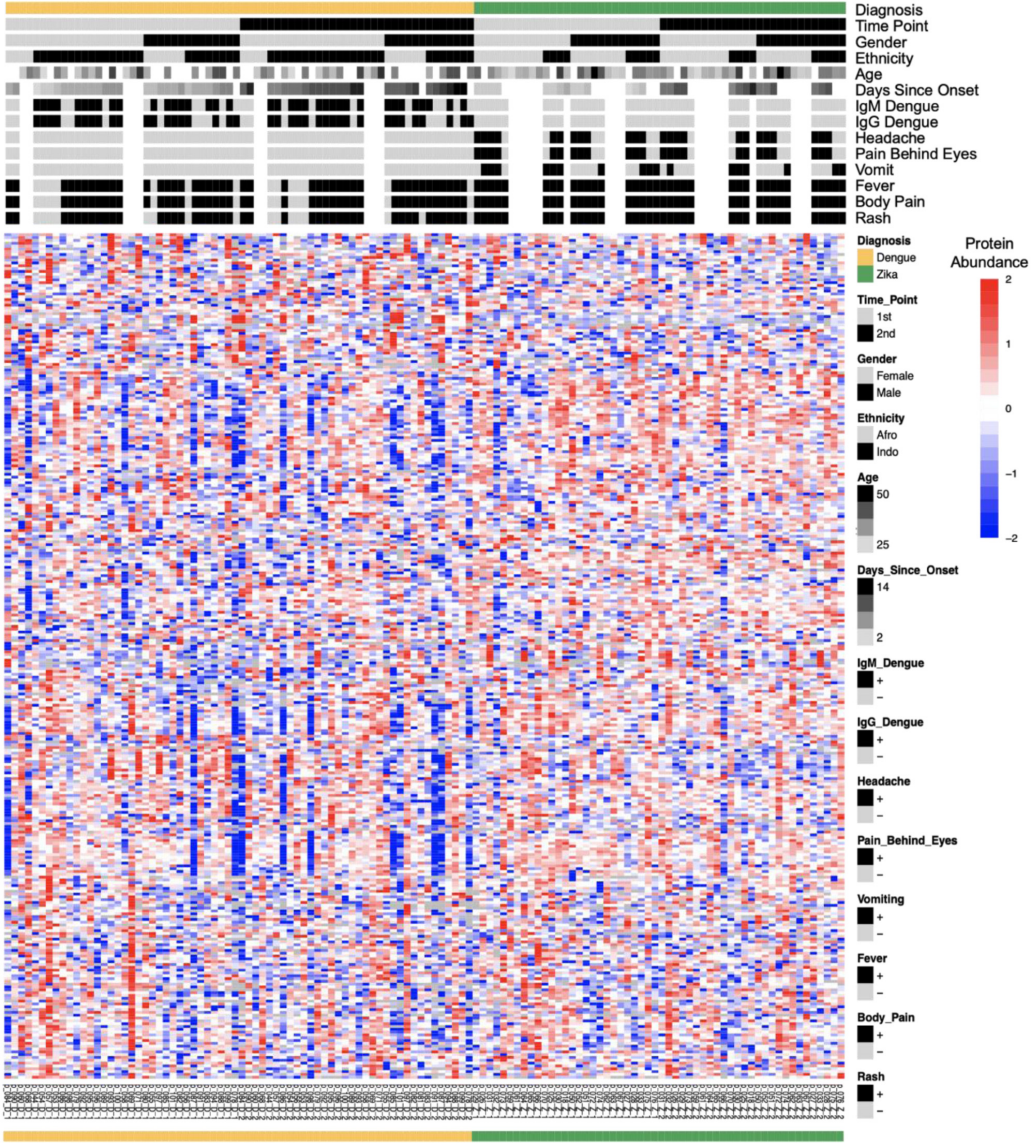
**Quantitative proteomics reveals diverse expression patterns across patients.** The heatmap shows the normalized, log base 2 transformed and row-wise scaled protein abundances derived from mass spectrometry. The corresponding meta-data is shown above: diagnosis, sample time point 1 and 2, gender, ethnicity (Afro- and Indo-Trinidadian), age (years), number of days since onset of symptoms, positive (+) or negative (−) dengue IgM and IgG ELISA results, presence (+) or absence (−) of headache, pain behind the eyes, vomiting, fever, body pain, and rash. Missing values in protein data are shown in *white*.

To obtain a quantitative proteomic picture of the patient response to DENV and ZIKV infection, we used an integrated workflow to map 517 groups of indistinguishable protein isoforms across the 122 samples from the 2016/2017 cohort. The lead protein for each protein group provided the name for the group. The heatmap in Figure 2 presents these data for the 277 protein groups with high-confidence identifications (also see supplemental Table S2). Protein abundances ranged over five orders of magnitude, measured as intensity in the mass spectrum. The protein expression patterns were diverse across patients, but showed some biases toward diagnosis (patients with DENV *versus* ZIKV infections), gender, and time point of sample collection.

We examined these biases in patient characteristics in more detail through analysis of the principal components that mark variation in the protein expression matrix. The first five principal components explained 43% of the variation in total (Table 1, supplemental Table S3). The first principal component (PC) explained 21% of the expression variation, and PC scores of patients correlated significantly with diagnosis (DENV/ZIKV, *p*-value = 0.002), indicating that most of the expression phenotype is indeed driven by the patient’s symptoms. The first component also correlated strongly with one clinical variable, “vomit,” suggesting it to be an indicator of ZIKV infection (*p*-value = 0.040). The other components correlated with both diagnoses, detection of the anti-DENV IgM and IgG biomarkers, and some clinical features, such as headache, pain behind the eyes, and vomit. These relationships confirmed that a biological signal could be detected in the data but also the presence of several additional factors.

**Table 1:** Variation in protein expression is driven by diagnosis and other clinical variables

Principal component (protein expression variation explained)	Diagnosis	Dengue IgM	Dengue IgG	Headache	Pain behind eyes	Vomit	Fever	Body pain	Rash	Gender	Age	Ethnicity	Days since onset
PC1 (21%)	0.00	0.23	0.77	0.10	0.10	0.04	0.95	0.95	0.87	0.17	0.88	0.45	0.33
PC2 (8%)	≤0.0001	0.00	0.01	≤0.0001	≤0.0001	0.08	0.63	0.63	0.90	0.76	0.84	0.61	0.96
PC3 (6%)	0.47	0.18	0.16	0.66	0.66	0.81	0.52	0.52	0.40	0.06	0.05	0.71	0.63
PC4 (4%)	0.87	0.27	0.56	0.12	0.12	0.09	0.76	0.76	0.86	0.89	0.76	0.60	0.48
PC5 (3%)	0.14	0.30	0.25	0.03	0.03	0.02	0.24	0.24	0.25	0.96	0.71	0.40	0.55

The first five principal components from a protein-level Principal Component Analysis were correlated with patient meta-data in a linear regression model. The table shows resulting *p*-values with significant ones (*p* ≤ 0.05) highlighted in red. The dengue/Zika diagnosis correlates with principal components (PCs) 1 and 2, positive/negative anti-DENV IgM and IgG results with component 2, the presence/absence of symptoms headache and pain behind the eyes with components 2 and 5, and the presence/absence of vomiting with components 1 and 5. The three symptoms—headache, pain behind the eyes, and vomiting—were exclusively diagnosed in patients with ZIKV infection. Further symptoms, as well as gender, age, ethnicity, and number of days since onset of symptoms, did not correlate with any of the first five principal components. See supplemental Table S4 for correlation of principal components at fragment level, which confirm these results.

### Thirteen Proteins Are Significantly Differentially Expressed

**Fig. 3:**
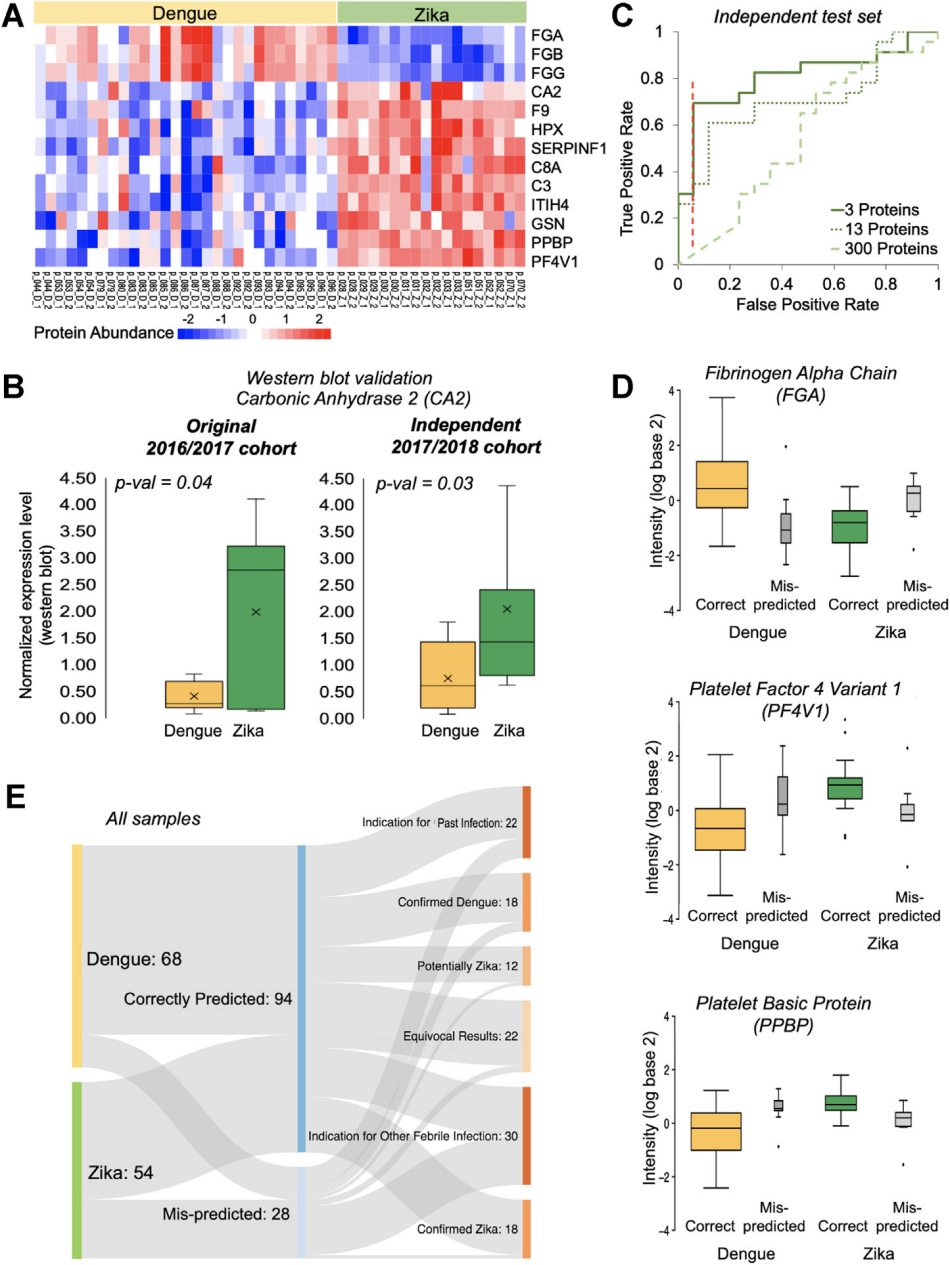
**Statistical modeling identifies signatures of differentially expressed proteins.***A*, two-pronged modeling identifies 13 proteins that are significantly differentially expressed between patients with DENV and ZIKV infection (adjusted *p*-value ≤ 0.05). The heatmap shows the row-wise clustered and scaled residual abundance data after removing confounding effects for the 47 samples with complete meta-data. The extended abundance data for the 13 proteins is shown in supplemental Table S3. *B*, western blotting confirms differential expression for Carbonic anhydrase II (CA2) for patient samples from both the original cohort (2016/2017) used for the proteomics analysis and a new, independent cohort (2017/2018). Not shown: one extreme value for Zika (2017/2018 cohort) with normalized expression of 6.82. Anti-CA2 western blot signals were normalized by signal of reference Ponceau staining. Crosses within the box-and-whiskers plots indicate the average. *p*-values were calculated for two-tailed *t* test of normalized expression values of dengue *versus* Zika samples within a cohort. Full images are shown in supplemental Fig. S8. Quantitation is provided in supplemental Table S7. *C*, using the 13 differentially expressed proteins and a subset of three most predictive proteins, we evaluated model performance on an independent test set. For the classification with three most predictive proteins, we obtained 70% true-positive Zika predictions at a 6% false-positive rate (indicated by *red dashed line*). The area underneath the receiver–operator curve is 0.81. *D*, correctly predicted samples with DENV and ZIKV infections differ in the intensity (log2 transformed) of the three best proteins. Mispredicted samples have intermediate intensities. *E*, our predictions are confirmed by outside evidence. Additional experimental testing on a subset of samples (supplemental Table S6) revealed cases of ambiguous diagnosis: samples with equivocal results, samples of dengue diagnosis, which might be Zika infections or which may also have signs of past infections, and some Zika samples with an indication for other febrile infections. The graph evaluates our predictions with respect to the original diagnosis and these additional findings. Supplemental Fig. S5 shows this graph for the test set only.

Next, we extracted proteins differentially expressed between patients diagnosed with DENV and ZIKV infections. To do so, we first divided the data into a training and test set and then used a two-pronged approach in which we evaluated differential expression without and with consideration of confounding factors (Fig. 1, Methods). The union of these two approaches identified 13 proteins as significantly differentially expressed (adjusted *p*-value < 0.05) (Fig. 3*A*). Most of these proteins were expressed at higher levels in ZIKV than in DENV infections. Notably, the protein expression levels still vary substantially across patient samples.

Table 2 describes the 13 proteins in their biological roles and known relationships to DENV and ZIKV infections, as well as pregnancy and brain function. Supplemental Table S5 provides extended descriptions. As expected for serum samples from virus-infected patients, most proteins identified were involved in the immune response and wound repair, as is common in febrile syndromes. Many of the proteins have known relationships to either DENV or ZIKV infections: for example, three of the differentially expressed proteins have also been identified in an independent proteomic study comparing patients with DENV infection with healthy controls (C3, HPX, and ITIH4) (20). Further, with just one exception, all 13 proteins had some relationship to pregnancy, often related to pre-eclampsia, and regulation of brain function (Table 2, supplemental Table S5). While statistically significant, most fold-changes (expression differences) between samples from DENV and ZIKV infections are very small, *i.e.*, less than 1.5-fold (supplemental Table S5, supplemental Fig. S4), with the exception of fibrinogens, PF4V1, and Carbonic Anhydrase 2 (CA2), which show up to threefold differences in expression levels between the two sample types.

**Table 2:** Differentially expressed proteins present signatures of each infection

Gene	Protein	Function	Up or down in patients with ZIKV compared with DENV infection	Relationship to			
				Dengue	Zika	Pregnancy	Brain
C3, C8A	Complement Components (C3, C8A)	Protection of the host from infection/inflammation	up	yes	yes	yes	yes
CA2	Carbonic anhydrase II	Reversible hydration of carbon dioxide	up	n/a	n/a	yes	yes
F9	Coagulation factor IX	Blood coagulation	up	yes	n/a	yes	n/a
FGA, FGB, FGG	Fibrinogen (alpha, beta, gamma chain)	Hemostasis as one of the primary components of blood clots	down	yes	yes	yes	yes
GSN	Gelsolin	Calcium-regulated, actin-modulating protein	up	n/a	n/a	yes	yes
HPX	Hemopexin	Hemopexin binds heme and transports it to the liver	up	yes	n/a	yes	yes
ITIH4	Inter-alpha-trypsin inhibitor heavy chain H4	Inflammatory responses	up	yes	yes	yes	yes
PF4V1	Platelet factor 4 variant 1	Inhibitor of angiogenesis and an inhibitor of endothelial cell chemotaxis *in vitro*	up	yes	yes	yes	yes
PPBP	Pro-platelet basic protein	Chemoattractant and activator of neutrophils to stimulate various cellular processes	up	yes	yes	yes	yes
SERPINF1 (PEDF)	Pigment epithelium-derived factor	Induces extensive neuronal differentiation, inhibitor of angiogenesis	up	n/a	n/a	yes	yes

The table shows the 13 differentially expressed proteins (FDR ≤0.05) and their known connections to DENV and ZIKV infections as well as to brain and pregnancy-related studies. Supplemental Table S3 shows extended descriptions and literature references for the respective proteins.

We validated the results using western blotting from both the original 2016/2017 and an independent cohort of patients diagnosed with DENV or ZIKV infections collected in 2017/2018 (Fig. 3*B*). We selected proteins for validation based on the expected expression difference between DENV and ZIKV samples (supplemental Table S5) and antibody availability. Indeed, CA2 shows higher expression levels in samples with Zika compared with those with dengue diagnosis (*p*-value < 0.05), consistent with the proteomics results for the 2016/2017 cohort (Fig. 3*A*). The expression difference is again small, but significant. While CA2 has not been linked to dengue or Zika virus infection (Table 2), there is evidence for its role in brain development and function (21), as well as respiratory-distress syndrome in infants (22). Antibodies for other proteins failed to provide reproducible western blot results (Methods).

### Extensive Feature Selection Finds a Protein Signature to Distinguish Between DENV and ZIKV Infections

Next, we extracted the proteomic profiles for the 13 differentially expressed proteins for all samples in the training data set and tested different algorithms in their ability to distinguish computationally between ZIKV and DENV infections (Methods). We evaluated the true- and false-positive rates of the best performing algorithm in the independent test set using the primary diagnosis made when the samples had been acquired in Trinidad (Fig. 3*C*). We also extracted a set of three “Best Proteins” whose predictive ability outperformed that of the 13 proteins even further (Fig. 3*C*). These proteins were FGA, PF4V1, and PPBP shown in Figure 3*D*. The set of three best proteins predicted ∼70% true-positive ZIKV infections at a false-positive rate of 6%. The overall area underneath the receiver–operator curve was 0.81.

We examined three best proteins in more detail to verify their putative roles during the response to infection. The distributions of the proteins’ expression levels are shown in Figure 3*D*. FGA and other fibrinogens are components of blood clots and involved in early wound repair; and perturbation in coagulation has been linked to both Zika and dengue infections (23, 24, 25, 26). FGA is one of the few proteins with lower expression levels in ZIKV compared with DENV infections, although plasma fibrinogen concentrations are known to be repressed in patients with DENV infections compared with control (27). Furthermore, DENV and its antibodies can directly influence the fibrinolytic pathway, providing further support for FGA being differentially expressed (28). In comparison, it has been shown for at least one patient with Zika diagnosis that the infection can lead to severe liver injury and coagulation disorders with altered blood levels of FGA and other fibrinogens (29). Further, FGA stabilizes embryonic-placental attachment during pregnancy, but may have additional roles in embryonic development (30, 31). Fibrinogen supports brain function through remyelination of the nervous system (32, 33). However, it is too early for our results to provide mechanistic insights into complications experienced by pregnant ZIKV-infected patients, largely due to the lack of healthy controls in the cohort analyzed here.

PF4V1 is an inhibitor of angiogenesis and is expressed at higher levels in patients with ZIKV than with DENV infections (Fig. 3*D*). Consistently, it has been found downregulated in platelets from patients with DENV infections compared with control (34), as high levels can promote rapid replication and propagation of the virus (35). Indeed, the platelet count is one of the diagnostic indices used to distinguish between DENV and ZIKV infections (36).

PPBP is a platelet-derived growth factor that belongs to the CXC chemokine family and, as for PF4V1, is expressed at higher levels in patients with ZIKV than with DENV infections (Fig. 3*D*). Consistent with its function as an activator of neutrophils, it has been identified as part of an 18-gene signature of severe DENV-infected patients with secondary, potentially ZIKV coinfection (37).

Next, after combining the predictions for training and test sets, we examined the mispredictions in more detail, using data from additional serological testing with anti-ZIKV IgM and IgG, which confirmed or modified the original diagnosis (Fig. 3*E*, supplemental Table S6). Overall, the model predicted diagnosis correctly for 94 samples and incorrectly for 28 samples (mispredictions). Our approach correctly classified 17 of 18 ZIKV samples for which diagnosis was confirmed by additional testing. For DENV, 15 out of 18 confirmed samples had been correctly predicted. Of the 28 mispredicted samples, the additional serological testing revealed evidence for past/other febrile infections, potentially incorrect primary diagnosis, and equivocal results for 24 of the samples. This result indicates that mispredictions may result not only from model insufficiencies, but also from the complex nature of infections.

### Time-Resolved Proteomics Illustrates Patterns for Patients with Potential Past Infections

Finally, we examined the samples with ambiguous diagnosis, *i.e.*, samples with evidence for other infections or with equivocal results, for specific protein signatures. To do so, we applied our computational pipeline to the sample set, but labeled the samples as arising from either clear or ambiguous diagnosis. We emphasize that interpretation of these shared protein signatures is speculative and requires further investigation.

We defined clear diagnosis as those cases that were confirmed by additional serology (supplemental Table S6). We defined ambiguous diagnosis for two patient groups. First, we applied the definition to patient samples with an original dengue diagnosis, but positive additional testing for anti-ZIKV IgG or IgM in at least one time point. In total, 21 samples from our training set of 80 samples qualified. Second, we applied the definition to patient samples with an original Zika diagnosis but with negative results in the additional serological testing; this applied to 22 samples from our training set. Note that each group of ambiguous cases likely contains several false-positives arising from cross-reactivities of the respective antibodies.

We then used the two-step analysis pipeline to identify significant differential expression between the samples with clear and ambiguous diagnoses. The combined output resulted in nine proteins with *p*-values <0.01 (supplemental Fig. S4). Note that none of the events remained significant after multiple hypothesis correction, illustrating lack of statistical power in this cohort. Three of the factors were expressed at higher levels in ambiguous than in unambiguous cases—AHSG, TFRC, DHX9—and are involved in inflammation, innate immunity, and immunodeficiency, which are components of past infections (38, 39, 40, 41).

**Fig. 4:**
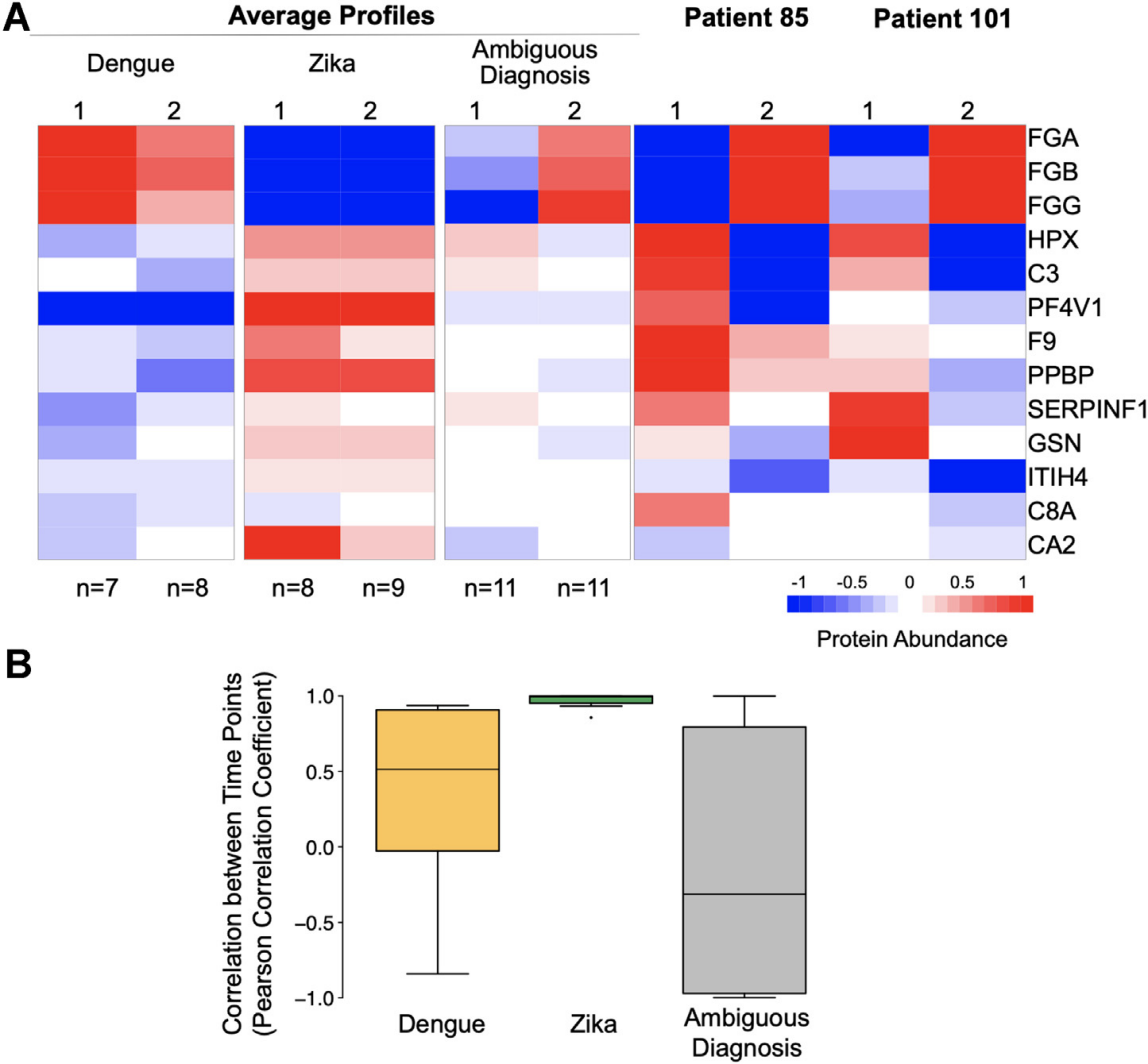
**Potential cases of past infections show distinct expression changes between consecutive time points.***A*, the average DENV and ZIKV infection profiles show strong expression differences among the 13 differentially expressed proteins and high similarity between the two measurement time points. These expression differences are not observed for the average profiles of samples with evidence for coinfection as specified by the additional serological testing (supplemental Table S6). Note that we do not have direct evidence for dual infections. When plotting individual patient samples with ambiguous diagnosis (Patient 85 and Patient 101), expression differences become apparent: within approximately 1 week between the first and second measurement point, both patients switch from a ZIKV infection-like to a DENV infection-like expression pattern. *B*, this trend is confirmed when examining average correlation of expression profiles for the three best proteins from the two time points across groups of patients: patients with clear ZIKV diagnosis show a much higher temporal correlation in protein expression than patients with an ambiguous diagnosis. Correlation profiles for patients with clear DENV diagnosis are more heterogeneous over time, but still more correlated than the ambiguously diagnosed patients (“Indication for Co-Infection”). Supplemental Fig. S6 shows the results for all 13 differentially expressed proteins, confirming this trend. Seven out 144 data points were imputed using nearest neighbor averaging. DENV: n = 5, ZIKV: n = 8; Ambiguous diagnosis (indication for coinfection): n = 10.

Further, we aimed at identifying temporal signatures of past infections as indicated by the additional serological testing (supplemental Table S6). To do so, we examined the respective cases for expression changes across the two measurement time points. We first compiled average protein expression profiles of the correctly predicted samples with DENV or ZIKV infections, which had been confirmed by additional serological testing (Fig. 4*A*, supplemental Table S6). These average profiles showed only small changes in protein expression between the two time points, but distinct differences in expression levels between DENV and ZIKV infection. Each infection had a unique signature. We then calculated the average protein expression profiles for patients with an indication of past infections (Fig. 4*A*, ambiguous diagnosis). The average expression levels were very similar across proteins or between time points, without distinct differences.

However, when we examined individual patients with positive serological test results for both DENV and ZIKV infections in at least one time point, such as patients 85 and 101, we observed a striking trend (Fig. 4*A*). Neither of the two patients showed “vomit,” “pain behind the eyes,” or “headache” as a symptom, which are exclusive characteristics for acute ZIKV infection, and they had both been originally diagnosed with DENV infection at the time of sample collection, testing positive for anti-DENV IgM and IgG antibodies at both time points. Additional serological testing showed positive results for anti-ZIKV IgM antibodies. Indeed, both patients showed protein expression patterns similar to that of Zika at the first time point, in contrast to the original diagnosis. Only in the samples from the second time point—collected about 7 days later—the protein expression patterns were similar to that of unambiguously diagnosed DENV infections. The change in expression was particularly striking for FGA, HPX, and C3. Both patients may have had previous ZIKV infections, which affected the response to the acute DENV infection with respect to temporal switch in protein expression profiles. This interpretation is consistent with DENV and ZIKV outbreaks in the region at the time of, and prior to, sample collection (Methods).

Finally, we confirmed the temporal disconnect between expression patterns for the entire set of patients with positive test results for both DENV and ZIKV infections (Fig. 4*B*). To do so, we examined the correlation between the protein expression signatures of the consecutive measurement time points for each patient. The correlation of expression values was low for patients with evidence for past infections and much higher for unambiguously diagnosed DENV or ZIKV infections (Fig. 4*B*). Therefore, we hypothesize that the response to an acute infection (onset of symptoms) may at first be dominated by the signature characteristic for past infections, but then switch to countering the current virus.

## Discussion

Our study provides a unique proteomic resource of serum samples from a cohort of 62 patients diagnosed with DENV or ZIKV infection. We quantified >500 proteins across all samples, with the complete, high-quality data set consisting of 277 proteins. Using a statistical analysis to remove the effects of the heterogeneity in the meta-data and sample collection, we identified proteomic signatures of the two types of infection. We extracted 13 differentially expressed proteins, most of which have links to pregnancy and brain function (adjusted *p*-value ≤ 0.05, Fig. 3).

We validated the differentially expressed proteins through both comparison with the literature and western blotting. Three of the 14 proteins had been identified in serum proteomes from DENV-diagnosed patients compared with healthy controls in India (20): Complement C3, Hemopexin, and Inter-alpha-trypsin inhibitor heavy chain H4. Two of the differentially expressed proteins were linked to the complement system (C3, C8A). The complement system has an ambivalent role in Flavivirus infection: it can be protective by limiting viral replication, or contribute to disease severity when excessively activated, and causing an exacerbated inflammatory response (42). Its occurrence among the differentially expressed proteins might link to the increased activity of the complement systems in DENV infections (43, 44). Using western blotting, we confirmed that CA2 is differentially expressed in DENV *versus* ZIKV patient samples in both the original and an independently collected cohort of samples (Fig. 3*B*).

Further, we used a machine learning approach to distinguish between the DENV and ZIKV infections based on protein expression profiles of the 13 differentially proteins and a subset of three proteins (FGA, PF4V1, PPBP). We achieved high sensitivity and specificity, *i.e.*, 70% true-positive Zika identifications at a 6% false-positive rate. These results outperformed other predictions based on the presence of viral proteins in blood samples (13). From the differentially expressed proteins, we extracted three “Best proteins”—FGA, PFV4F1, and PPBP—which occurred at the intersection of these predictive approaches. A review of literature confirmed the differential abundance of some of these proteins: PFV4F1, for example, has strong links to DENV propagation (35). Further, all three proteins showed links to pregnancy and brain function, which could help explain adverse effects of ZIKV infections.

Finally, we investigated the proteomic data for signatures of possible past infections with Zika, dengue, or other viruses. Some geographical areas have a high presence of both Flaviviruses (45), and Trinidad—the origin for the cohort analyzed here—witnessed a DENV outbreak in 2015/2016, simultaneously with reports on increased ZIKV occurrences in 2016/2017 (http://paho.org). To identify possible proteomic signatures of past infections, we divided the cohort into unambiguously diagnosed patients and those with ambiguous test results. We identified nine proteins with significant differential expression (*p*-value < 0.01, supplemental Fig. S5). Among these proteins was Fetuin-A (Alpha-2-HS-glycoprotein), which had higher expression levels in cases of ambiguous as compared with clear diagnosis. Fetuin-A has not only been identified as an interactor of DENV protein NS1 (46), but also is a known marker of inflammation during acute-phase infections (39, 47, 48). Its anti-inflammatory roles (49) might explain its higher levels in patients with ambiguous diagnoses as these samples are enriched for patients with prior infections and coinfections that stimulate inflammation. We identified proteomic patterns across the two measurement time points obtained for each patient: patients with unambiguous, single infections showed temporally consistent expression of the core proteins. In contrast, patients with mixed diagnoses showed clear temporal evolution in the protein expression profiles over the course of the ∼1 week between the two different time points.

While intriguing and based on rigorous statistical filtering, the proteomics data and results presented here serve as a resource to characterize the response to the different infections, rather than defining new biomarkers. Interpretation of the results presented here is entirely speculative. Part of the ongoing challenges are the heterogeneity of the samples and the small (albeit significant) expression differences observed, which would prevent use of the proteins as markers in a clinical setting. Future work will have to include larger cohort sizes, controlled time-dependent sampling, healthy controls, and additional independent experiments to support further interpretation.

## Data Availability

All data generated or analyzed in this study are included in this published article (and its supplementary information files). The source data underlying figures and supplementary figures is provided as a Source Data file. The mass spectrometry proteomics data, including the Spectronaut files, have been deposited to the ProteomeXchange Consortium *via* the PRIDE (50) partner repository with the data set identifier PXD015422. R scripts are deposited in github (https://github.com/cvogelnyu/DengueZikaProteomics).

## Supplemental data

This article contains supplemental data.

## Conflict of interest

The authors declare no competing interests.
