# Supplementary material for: New Proteomic Signatures to Distinguish Between Zika and Dengue Infections
Source: Mol Cell Proteomics. 2021 Feb 12;20:100052. doi: 10.1016/j.mcpro.2021.100052 (PMC8042398; doi:10.1016/j.mcpro.2021.100052)

Supplementary Notes

New proteomic signatures to distinguish between Zika and dengue infections

Authors:

Kristina Allgoewer 1,2*, Shuvadeep Maity 1*, Alice Zhao 1, Lauren Lashua 1, Moti Ramgopal 3, Beni N. Balkaran 4, Liyun Liu 5, Savita Purushwani 1, Maria T. Arévalo 5, Ted M. Ross 5,6, Hyungwon Choi 7, Elodie Ghedin 1,8**, Christine Vogel 1**

Affiliations:

1 New York University, Department of Biology, Center for Genomics and Systems Biology, New York, NY, USA

2 Humboldt University, Berlin, Germany

3 Martin Health System, Clinical Research Division, Stuart, FL, USA

4 University of the West Indies, Department of Clinical Medical Sciences, St Augustine, Trinidad & Tobago

5 University of Georgia, Center for Vaccines and Immunology, Athens, GA, USA

6 University of Georgia, Department of Infectious Diseases, Athens, GA, USA

7 Saw Swee Hock School of Public Health, National University of Singapore, Singapore

8 Department of Epidemiology, School of Global Public Health, New York University, NY, USA

*Co-first authors

**Corresponding authors: cvogel@nyu.edu, elodie.ghedin@nyu.edu

Supplementary Figures

Figure S1. Fragment intensity distributions before and after batch correction

A boxplot of log base 2 transformed fragment ion peak areas across all samples revealed a batch effect (top panel), probably caused by replacing the liquid chromatography column after 78 samples. Both sample time points of patient 48 were considered faulty and removed. Fragments with a high number of missing data points were removed by selecting those with ≥ 60% non-missing data across patient samples. The batch effect was corrected by fitting a lowess curve through each fragment and subtracting in each batch, then setting the average to a common overall average peak area value. Outliers (3 standard deviations) were removed. The resulting intensity distributions are uniform across samples (bottom panel).

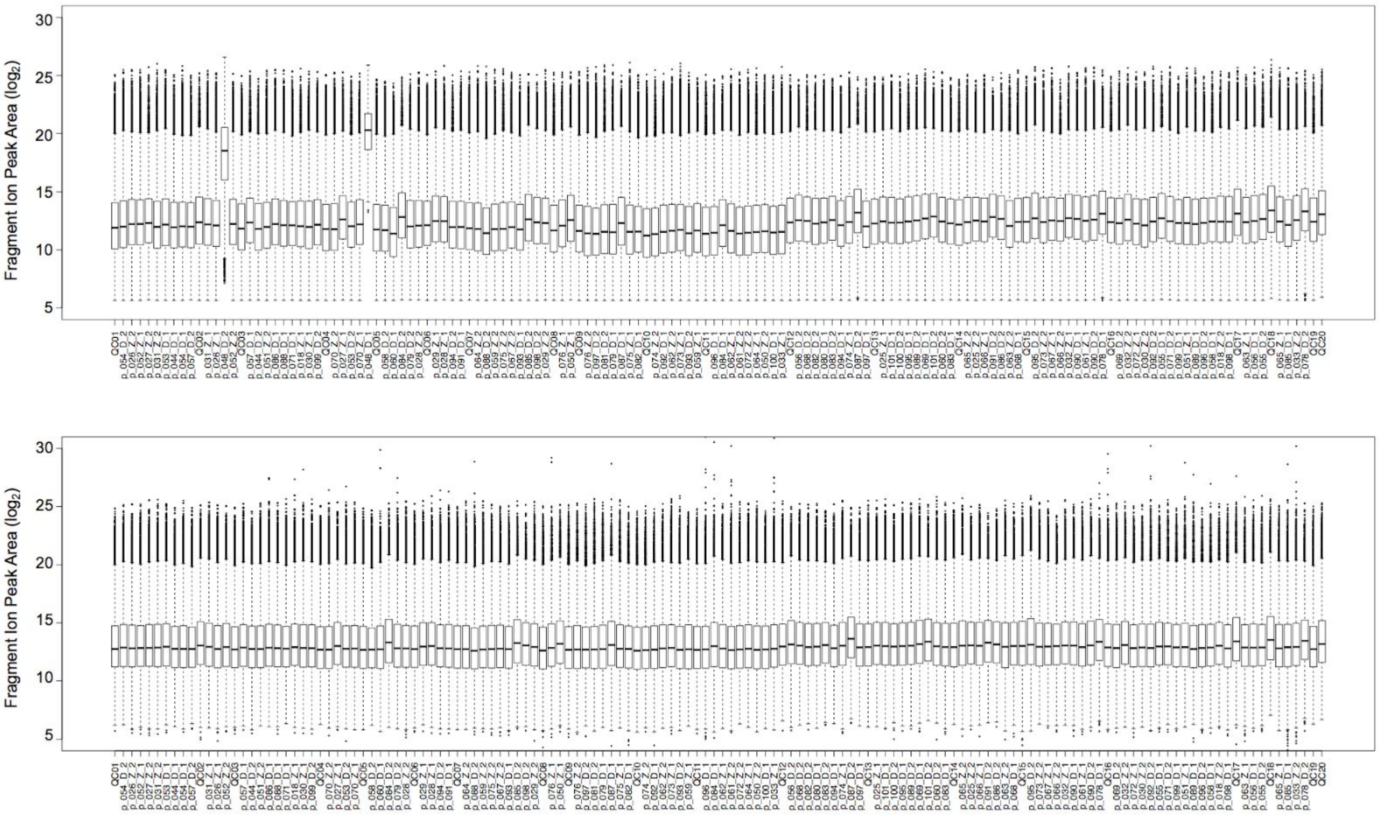

Figure S2. Coefficient of Variation in QC samples

We plotted the coefficient of variance (cov) of the abundance of all fragments detected in the Quality Control (QC) pooled samples. The median cov is at 26%. We did not apply a cov cutoff.

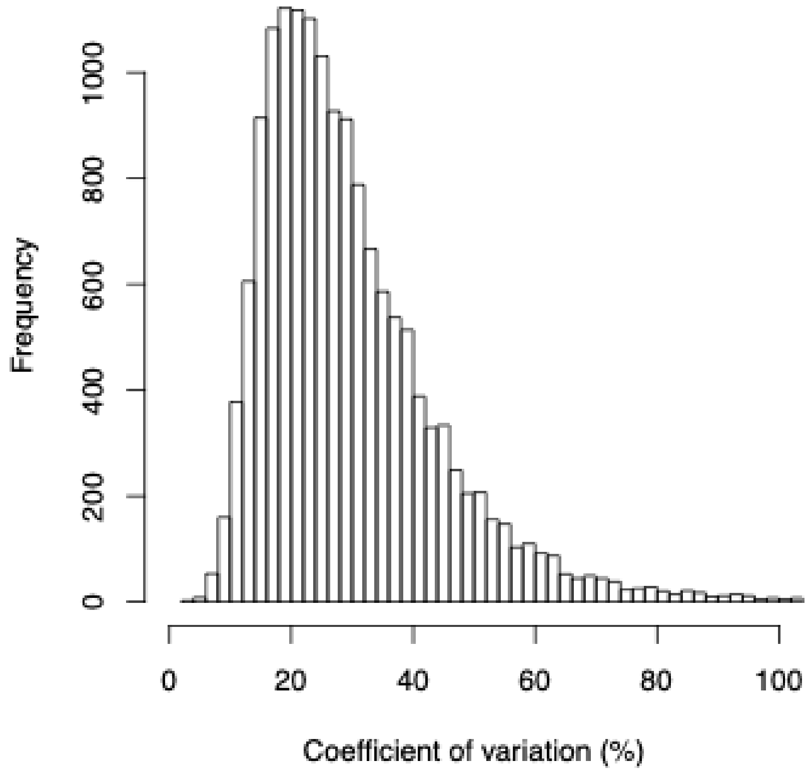

Figure S3. Effect sizes for the relationship between principal components (PCA analysis on protein level) and clinical and diagnostic variables with 90% confidence intervals (corresponds to 5% alpha levels).

Effect sizes are only shown for results with p-levels < 0.05. Since the only significant p-values were those from discrete variables (ANOVA results), we measured effect size as η^2 (eta squared) with: η^2 = sum of squares/(sum of squares + residual sum of squares). The guidelines for η^2 according to Cohen (1988) are: Small effect size: 0.01; Medium effect size: 0.059; Large effect size: 0.138. An η^2 of 0.1 also means that 10% of the change in the dependent variable can be accounted for by the independent variable.

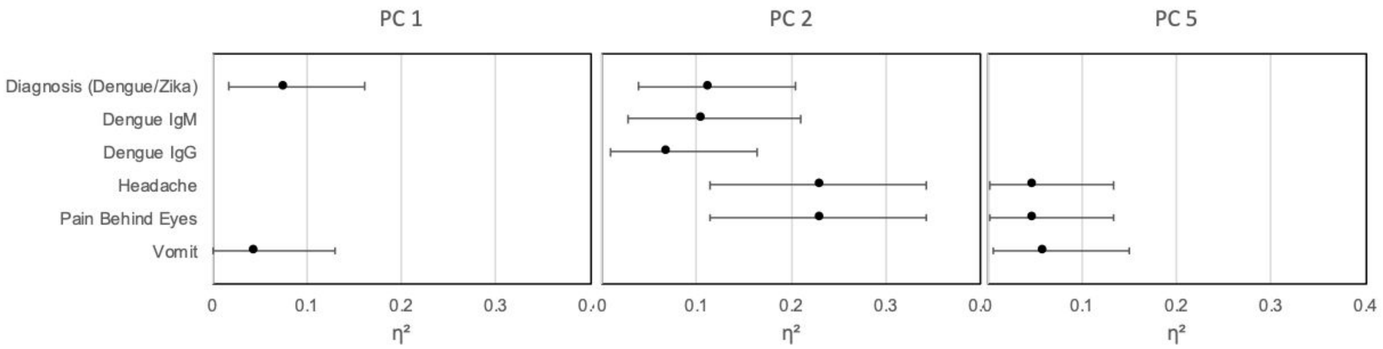

Figure S4. Abundance data for the 13 differentially expressed proteins

A. Heatmaps of normalized, row-mean centered, row-wise scaled and log base 2 transformed expression values for 13 differentially expressed proteins across all samples with unambiguous diagnosis. Missing values have been replaced by half the global minimal value. **B.** Box-and-whiskers plots of the distributions of the values from **A. C.** Intensity distributions for fragment ions for the two significantly differentially expressed proteins which had been quantified based on a single peptide (CA2 and FGG). For comparison, fragment intensities for a protein with single-peptide quantification that was not significantly differentially expressed (IGLV6-57).

A.

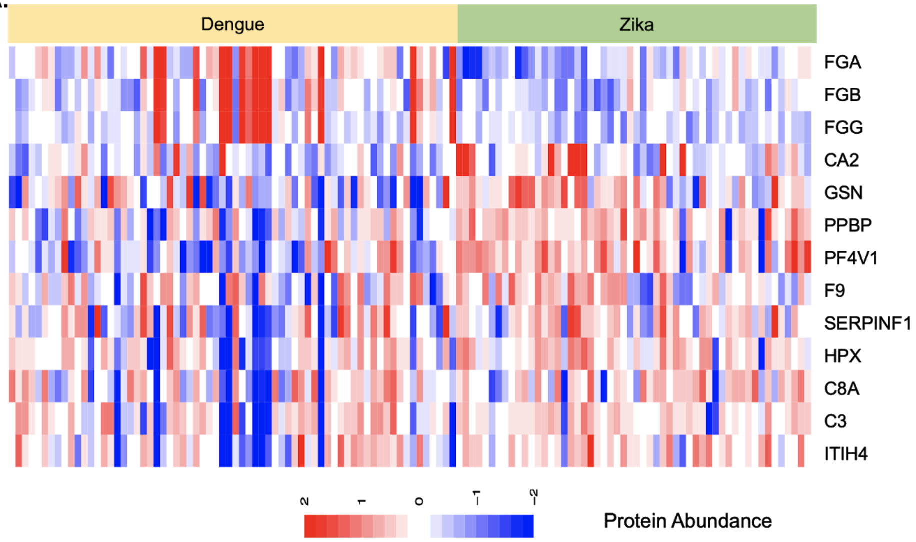

B.

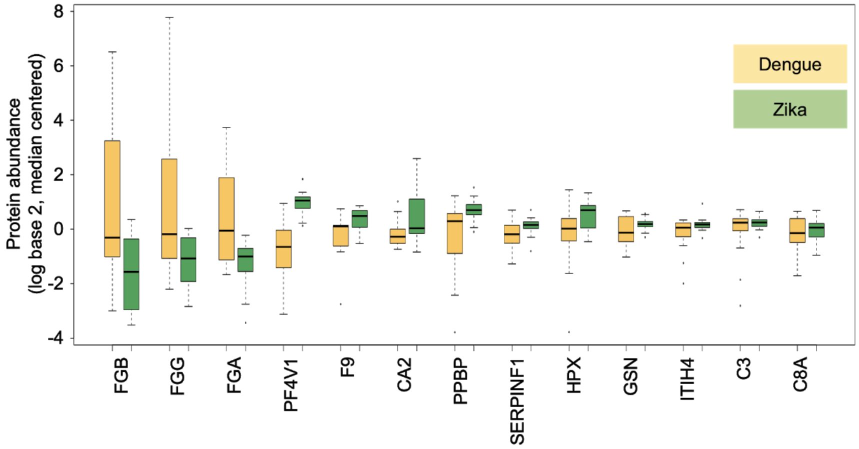

C.

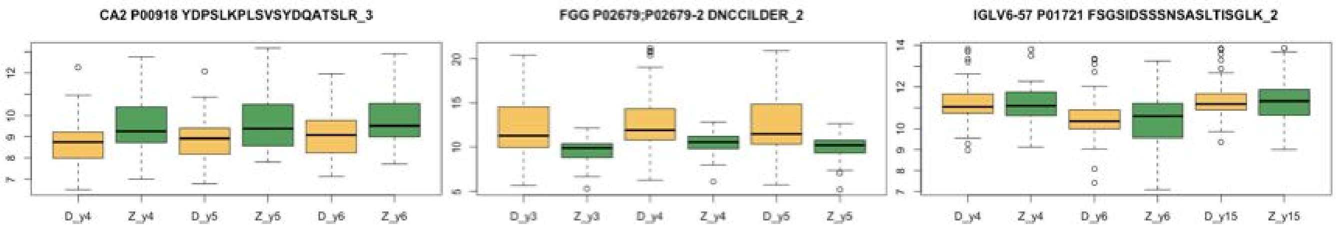

Figure S5. Predictions on test set only

The predictions were confirmed by outside evidence. Additional experimental testing on a subset of samples (**Supplementary Table S6**) revealed cases of ambiguous diagnosis: samples of dengue diagnosis with an indication of past infections, and some Zika samples with an indication for other febrile infections. The graph evaluates the predictions with respect to the original diagnosis and these additional findings for the samples in the test (evaluation) set.

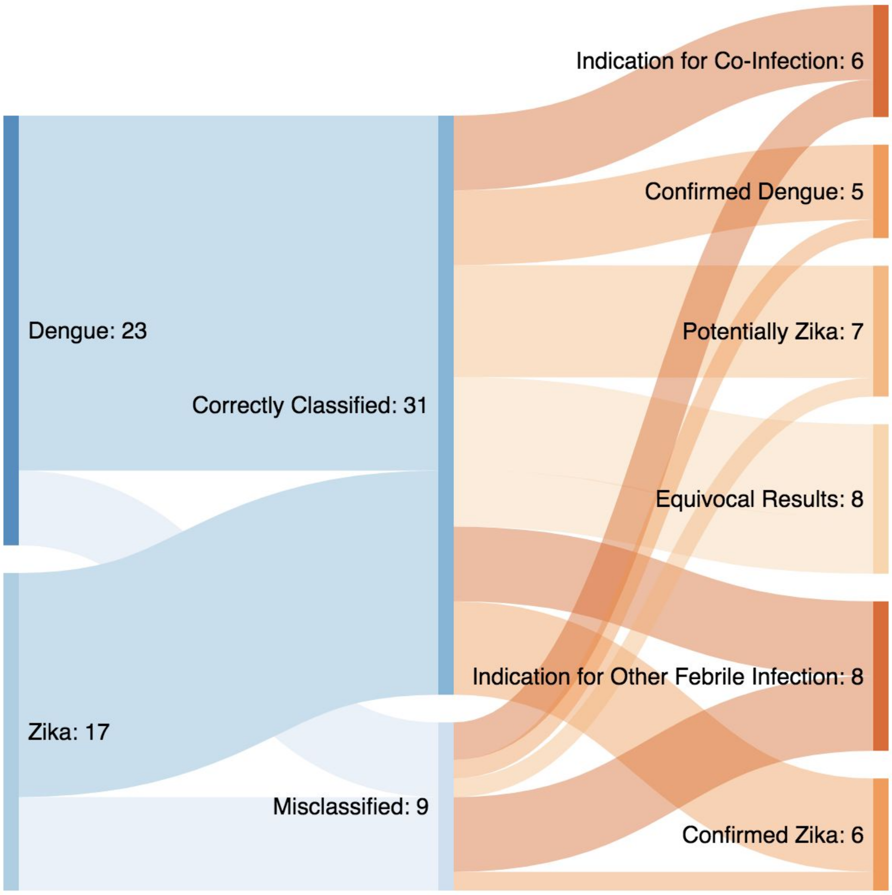

Figure S6. Correlation between protein abundances between different time points

For all 13 differentially expressed proteins, we calculated the Pearson Correlation Coefficient between measurements at time point 1 and time point 2 for confirmed cases of DENV and ZIKV infections, respectively, and patients from the category "Indication for Co-Infection" (or dual infection). Dengue: n=5, Zika: n=8; Ambiguous diagnosis: n=10.

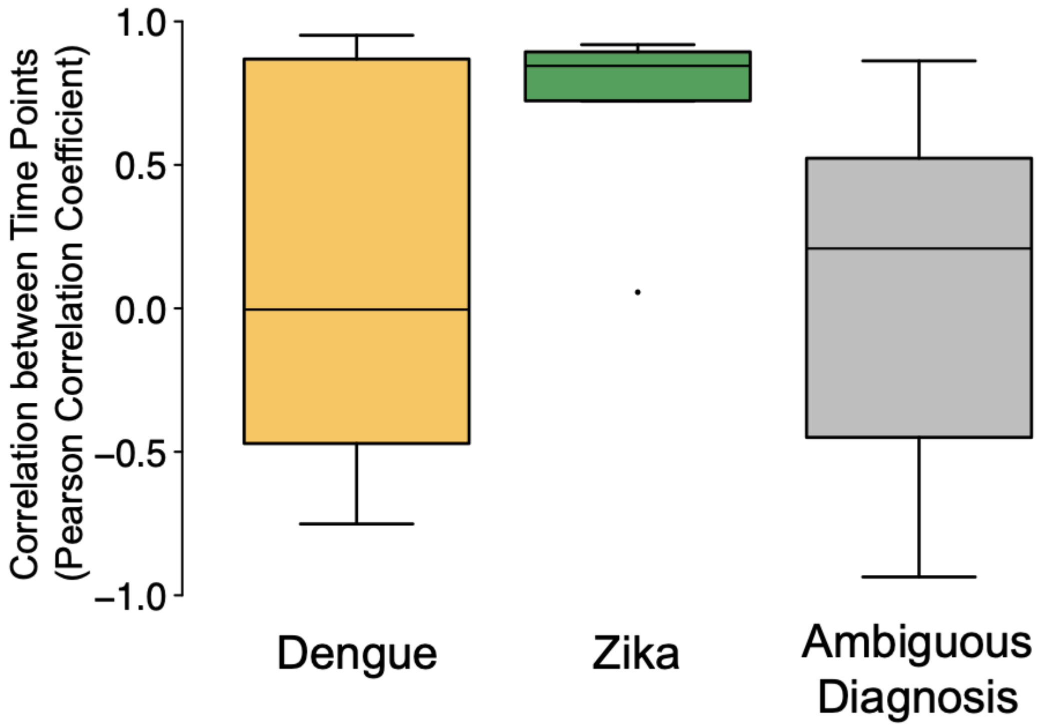

Figure S7. Proteins differentially expressed in samples with ambiguous diagnosis

We re-classified the 47 samples with complete meta-data based on the additional serological testing into those with confirmed (unambiguous) diagnosis and those with evidence for past infections (ambiguous diagnosis). **A.** Heatmap with residual data from Analysis B (47 samples with complete metadata). The heatmap shows the row-wise clustered and scaled residual abundance data after removing confounding effects. Using the same pipeline to remove confounding factors, we identified 2 proteins with expression levels higher in unambiguous than in ambiguous cases (THBS1, VTI1B) and 4 proteins with the opposite expression pattern (AHSG, DHX9, IGLC2, TFRC). Future work can test if these proteins indeed mark ambiguous diagnosis. **B.** Boxplot with original data (whole training set, 82 samples). **C.** Brief function description of the proteins taken from Uniprot (https://www.uniprot.org/).

A.

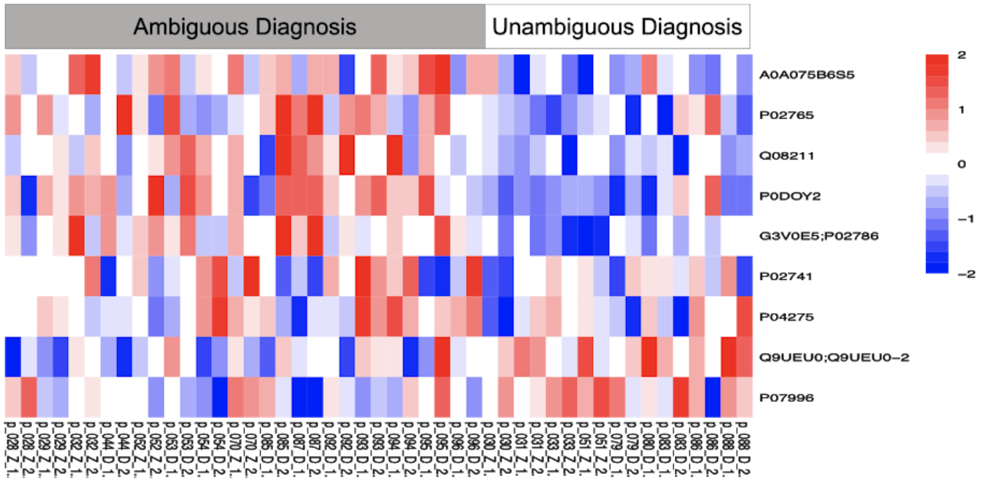

B.

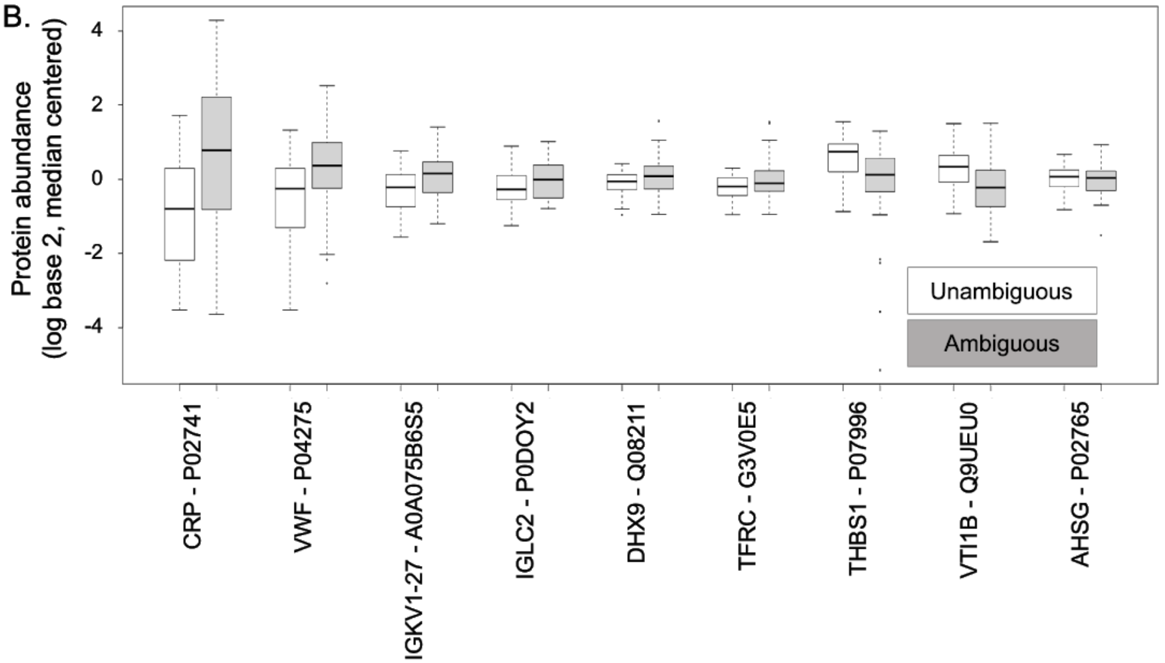

C.

Name	Function
CRP	C-reactive protein - Displays several functions associated with host defense.
VWF	von Willebrand factor - Maintenance of hemostasis, promotes adhesion of platelets to the sites of vascular injury
IGKV1-27	Immunoglobulin kappa variable 1-27 = V region of the variable domain of immunoglobulin light chains - participates in antigen recognition
THBS1	Thrombospondin-1 - Mediates cell-to-cell and cell-to-matrix interactions, binds heparin, bay play a role in dentinogenesis and/or maintenance of dentin and dental pulp
AHSG	Alpha-2-HS-glycoprotein - Promotes endocytosis, possesses opsonic properties and influences the mineral phase of bone
DHX9	ATP-dependent RNA helicase A - Unwinds DNA and RNA in a 3' to 5' direction, important roles in many processes, such as DNA replication, transcriptional activation, post-transcriptional RNA regulation, mRNA translation and RNA-mediated gene silencing
IGLC2	Immunoglobulin lambda constant 2 - Constant region of immunoglobulin light chains, antibody, membrane-bound or secreted glycoproteins produced by B lymphocytes
TFRC	Transferrin receptor protein 1 - Cellular uptake of iron occurs via receptor-mediated endocytosis of ligand-occupied transferrin receptor into specialized endosomes.
VTI1B	Vesicle transport through with t-SNAREs homolog 1B - Mediates vesicle transport pathways through interactions with t-SNAREs on the target membrane.

Figure S8. Western blot validation

We tested antibodies against FGA, FGG, and CA2. Only anti-CA2 antibodies provided consistent signals against samples from both the original 2016/2017 and the new 2017/2018 cohort. Unspecific binding of antibodies is typical for serum samples. We identified the CA2 band based on the molecular weight marker shown in the corresponding Ponceau stain. As serum samples do not provide consistent actin or tubulin for normalization, we used reference areas from the Ponceau stain for normalization of expression values. Each panel shows for a set e uncut Ponceau and western blot images as well as the cut images. **A.** Original 2016/2017 cohort for which the proteomics analysis had been performed. **B.** Independent 2017/2018 cohort from the same site with randomly chosen patient samples. Quantitation is provided in **Supplementary Table S7.** Uncropped western blot images are provided in the Source Data file.

A. 2016/2017 cohort

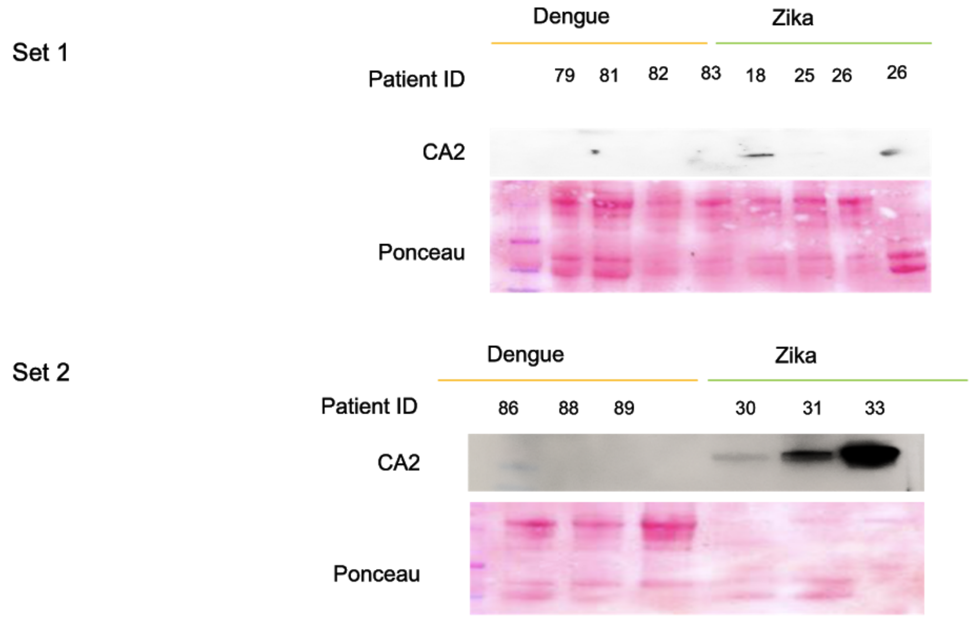

B. 2017/2018 cohort

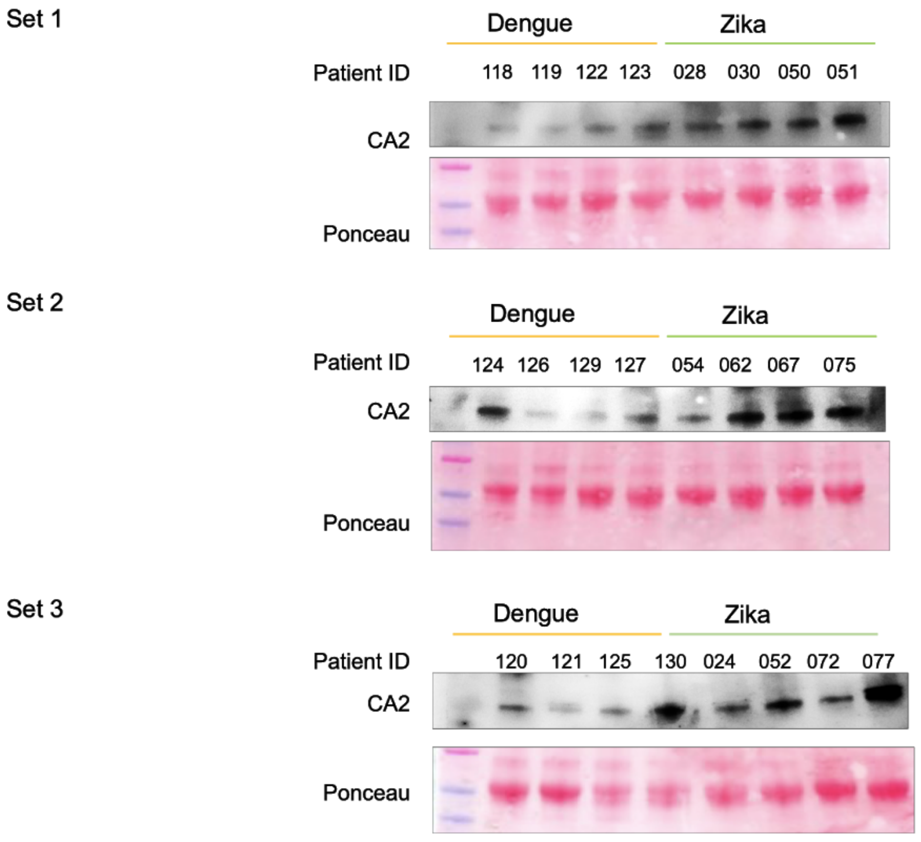

Supplement: Supplemental Figures S1–S8 [file mmc2.pdf]
